# Large-scale all-optical dissection of motor cortex connectivity shows a segregated organization of mouse forelimb representations

**DOI:** 10.1016/j.celrep.2022.111627

**Published:** 2022-11-08

**Authors:** Francesco Resta, Elena Montagni, Giuseppe de Vito, Alessandro Scaglione, Anna Letizia Allegra Mascaro, Francesco Saverio Pavone

**Affiliations:** 1 European Laboratory for Non-linear Spectroscopy, University of Florence, 50019 Sesto Fiorentino, Italy; 2 Department of Physics and Astronomy, University of Florence, 50019 Sesto Fiorentino, Italy; 3 Department of Neuroscience, Psychology, Pharmacology and Child Health (NEUROFARBA), University of Florence, 50139 Florence, Italy; 4 Neuroscience Institute, National Research Council, 56124 Pisa, Italy; 5 National Institute of Optics, National Research Council, 50019 Sesto Fiorentino, Italy

**Keywords:** RFA, CFA, motor cortex organization, motor mapping, grasping, *in vivo*, optogenetics, calcium imaging, wide-field microscopy, jRCaMP1a, ChR2

## Abstract

In rodent motor cortex, the rostral forelimb area (RFA) and the caudal forelimb area (CFA) are major actors in orchestrating the control of complex forelimb movements. However, their intrinsic connectivity and reciprocal functional organization are still unclear, limiting our understanding of how the brain coordinates and executes voluntary movements. Here, we causally probe cortical connectivity and activation patterns triggered by transcranial optogenetic stimulation of ethologically relevant complex movements exploiting a large-scale all-optical method in awake mice. Results show specific activation features for each movement class, providing evidence for a segregated functional organization of CFA and RFA. Importantly, we identify a second discrete lateral grasping representation area, namely the lateral forelimb area (LFA), with unique connectivity and activation patterns. Therefore, we propose the LFA as a distinct forelimb representation in the mouse somatotopic motor map.

## Highlights

• A large-scale single-photon all-optical system is developed
• Movement-specific activation maps show a modular organization of the motor cortex
• Activity propagation analysis reveals movement-specific spatiotemporal patterns
• An independent lateral grasping representation area is identified

## Graphical abstract

**Figure:**
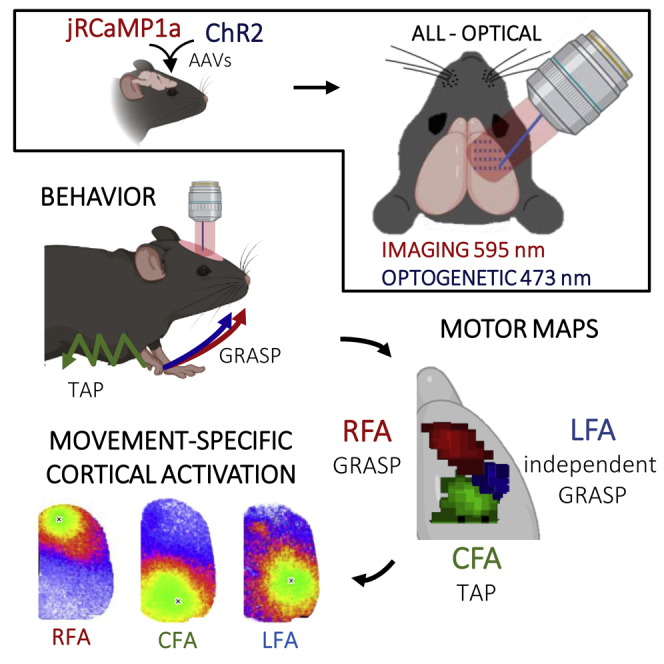

## Teaser

The intrinsic connections and functional structure of areas in the forelimb motor cortex are unclear. Here, Resta et al. develop an all-optical system to causally dissect cortical activation during movement execution, providing evidence for a segregated organization of RFA and CFA networks and identifying a second grasping representation area.

## Introduction

In rodents, forelimb movements are controlled by two distinct cortical functional areas: the caudal forelimb area (CFA) and the rostral forelimb area (RFA). These areas have been intensely investigated using mainly two different experimental paradigms: (1) the somatotopic mapping of the motor cortex, exploiting electrical or optogenetic stimulation, and (2) the recording of cortical activity during natural movement execution. Despite the extensive number of studies using these alternative approaches, a shared model of the motor cortex functional organization is missing (Morandell and Huber, 2017).

There are mainly two models that describe the functional relationship between CFA and RFA. The first model associates the RFA with the primate premotor area and the CFA with the primate M1, following a hierarchical organization (Ebbesen et al., 2018). This model is based on evidence demonstrating that RFA and CFA receive reciprocal functional projections generating corticocortical pathways involved in the transmission of forelimb sensory inputs, which seem to be processed first by the CFA (Kunori and Takashima, 2016). Moreover, it has been observed that RFA modulates CFA output, similar to how premotor areas affect motor areas in primates (Deffeyes, 2015). Conversely, the second model establishes that these two areas are parts of a highly integrated computational unit with distinct motor functions (Brown and Teskey, 2014). This model relies on evidence that RFA and CFA are characterized by two independent circuits, which lead to different motor outputs (Harrison et al., 2012; Hira et al., 2015; Karl and Whishaw, 2013). This hypothesis was further supported by studies showing that the output layers of these two areas have independent descending corticospinal projections (Baker et al., 2018; Economo et al., 2018).

Several loss-of-function experiments have been conducted to fill the gap between these models. Unfortunately, these approaches led to ambiguous results. On one hand, it has been demonstrated that simultaneous inactivation of RFA and CFA caused a deficit in movement execution (Kimura et al., 2017). Moreover, CFA inactivation did not significantly alter grasping in a task-related movement, whereas RFA inactivation was sufficient to strongly inhibit task performance. These results suggest that RFA plays a crucial role in forelimb movement (Kimura et al., 2017). On the other hand, it has been observed that movement execution deficits are exclusive to CFA inactivation, thus implying that RFA is functionally dependent on CFA activity (Morandell and Huber, 2017). Overall, these conflicting results have prevented the development of a complete model of motor cortex function.

The functional organization of the motor cortex is canonically investigated combining cortical electrical stimulation and behavioral readout to map cortical movement representations (Ramanathan et al., 2006; Tennant et al., 2011). However, the electrophysiological approach is invasive, is limited in spatial resolution, and lacks cellular population selectivity, thus it is being progressively replaced by optogenetics, which overcomes these limitations (Ayling et al., 2009; Hira et al., 2009). Light-based motor maps (LBMMs) are powerful tools to study motor cortex topography; however, important questions remain concerning the mechanisms that coordinate the activity of different functional areas during voluntary movement execution. To address this issue, we established a cross-talk-free large-scale all-optical experimental configuration combining wide-field fluorescence imaging of the red-shifted genetically encoded calcium indicator (GECI) jRCaMP1a and optogenetic stimulation of Channelrhodopsin-2 (ChR2) to perform light-based motor mapping of complex movements in awake mice while monitoring the mesoscale neuronal activity. Thanks to this low-invasive transcranial method, we investigated RFA and CFA effective connectivity and functional dependencies by causally dissecting the cortical activity patterns triggered by optogenetic stimulation. Our results show a modular organization of the movement-specific activated areas and activity propagation hallmarks during complex forelimb movement execution. Importantly, we identified a discrete lateral and caudal grasping cortical representation expressing distinct topographic and connectivity features.

## Results

### Wide and long-term stable cortical transfection of both jRCaMP1a and ChR2 in mouse motor cortex

**Figure 1:**
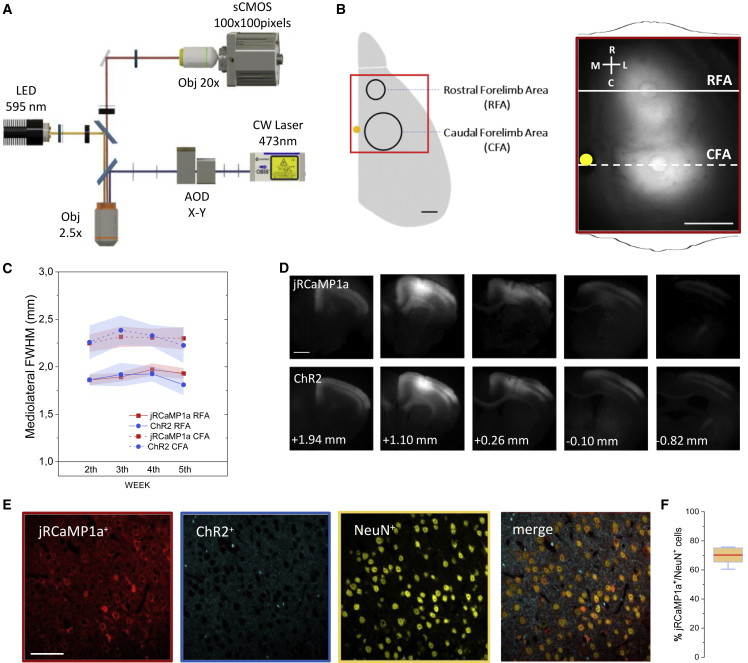
Experimental design to perform parallel functional imaging and light-based motor mapping in awake mice (A) Schematic representation of the double-path wide-field fluorescence microscope. (B) (Left) Schematic representation of the right cortical hemisphere and the CFA and RFA relative positions. Red square outline is the FOV of the wide-field fluorescence microscope. Scale bar, 1 mm. (Right) Example of the *in vivo* fluorescence spatial distribution of jRCaMP1a along the mediolateral plane passing through RFA and CFA. Yellow dot indicates bregma. Scale bar, 1 mm. White cross represents the rostrocaudal (R–C) and mediolateral (M–L) axes. (C) *In vivo* long-term quantification of jRCaMP1a (red) and ChR2 (blue) spatial distribution along the mediolateral plane passing through RFA (n = 7; solid line) and CFA (n = 7; dashed line) injection sites, as in (B). (D) *Ex vivo* coronal slices showing the rostrocaudal transfection extension of jRCaMP1a and ChR2. Scale bar, 1 mm. (E) Representative immunohistochemistry images showing the neuronal expression of jRCaMP1a (red), ChR2 (blue), and NeuN (yellow) in the motor cortex. Scale bar, 50 μm. (F) Quantification of the colocalization ratio jRCaMP1a^+^/NeuN^+^ (70.2 ± 4.9%, n = 7). Error bars represent SEM.

In mice expressing optogenetic actuators in the motor cortex, several classes of limb movements can be evoked depending on the stimulated site (Hira et al., 2009, 2015). To investigate large-scale cortical activity underlying optogenetically evoked movements, we infected the frontal right cortical hemisphere of C57BL/6 mice with adeno-associated viruses (AAV) carrying both the synapsin promoter-driven red-shifted GECI jRCaMP1a and the optogenetic actuator CaMKIIα promoter-driven ChR2. The optical setup consisted of a double-path illumination system integrated into a custom-made wide-field fluorescence microscope for parallel laser stimulation and wide-field cortical imaging (Figure 1A). To evaluate the transfection extension and its long-term stability, the spatial fluorescence intensity profiles were calculated (Figure 1B). In line with our previous observations (Montagni et al., 2019), we found that ChR2 and jRCaMP1a expression covered all the motor cortices, and their expression levels were highly stable over several weeks (Figures 1B and S2). Moreover, the expression profiles on histological brain slices showed that the transfection of jRCaMP1a and ChR2 was restricted to the motor cortex, reaching all the cortical layers (Figure 1D). In addition, to evaluate the synapsin-targeted jRCaMP1a transfection efficiency, brain slices were stained with the neuronal marker NeuN, and we detected neurons from both layer II/III and layer V, revealing that the jRCaMP1a^+^ neurons were 70.2% ± 4.9% of the NeuN^+^ cells (Figures 1E and 1F). In contrast, quantifying the ChR2 expression in neuron membranes was difficult, since ChR2-related fluorescence resulted in a diffuse widespread staining in the tissue, which made it problematic to identify the neuron’s shape, as also discussed in other papers (Miyashita et al., 2013; Watanabe et al., 2020). Our injection method leads to a wide transfection that covers all the motor cortices and is sufficiently stable to perform experiments several weeks after injection.

### Wide-field imaging of jRCaMP1a does not induce ChR2 cross-activation

The all-optical approach we chose to visualize cortical activation during optogenetically evoked complex movements combines blue-activated opsins and red-shifted GECIs (Akerboom et al., 2013; Dana et al., 2016; Farhi et al., 2019; Zhao et al., 2011). To date, there are two main red-shifted GECI families, comprising the RGECO and RCaMP variants based on mApple and mRuby protein, respectively. Despite RGECOs showing higher Ca^2+^ affinity and larger dynamic range than RCaMPs, they exhibit significant photoactivation when stimulated with blue light, thus hindering their combination with blue- and green-activated opsins (Akerboom et al., 2013). Indeed, most all-optical systems exploiting single-photon excitation critically suffer for cross talk between imaging and photostimulation (Fajardo et al., 2013; Lim et al., 2012). To assess the possible cross-activation of ChR2 during wide-field imaging of jRCaMP1a, we recorded the local field potential (LFP) during an alternated on/off pattern of the imaging illumination path (Figure S1A). Illumination-triggered average of the LFP showed no significant differences in the normalized power content of the standard neurophysiological spectral bands (Figure S1B). This result suggests that there were not relevant alterations of the neuronal activity during imaging. Therefore, we evaluated whether the laser wavelength used for optogenetic stimulation affected the jRCaMP1a readout. Single laser pulses at increasing intensity were delivered in mice expressing (jRCaMP1a^+^/ChR2^+^) or lacking the optogenetic actuator (jRCaMP1a^+^/ChR2^−^) (Figure S1C). The results showed a clear asymptotic increase in the jRCaMP1a response in jRCaMP1a^+^/ChR2^+^ mice. Conversely, laser pulses in jRCaMP1a^+^/ChR2^−^ mice did not induce jRCaMP1a responses up to 20 mW (Figure S1C). The results demonstrate that our all-optical configuration wards off the cross talk between imaging and photostimulation.

### Stereotyped cortical activation features of optogenetically evoked movements

**Figure 2:**
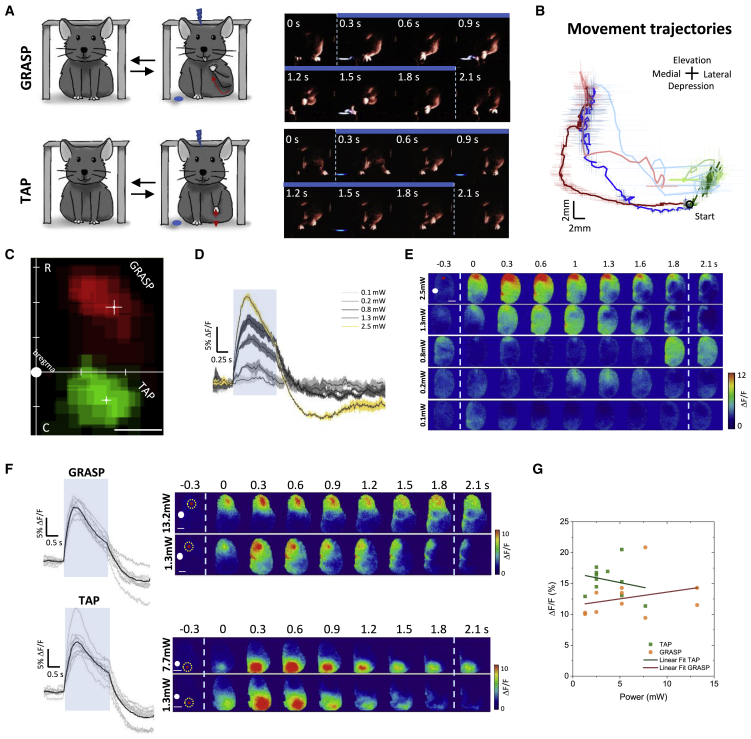
Stereotyped cortical activation features of optogenetically evoked movements (A) (Left) Representative cartoons describing the evoked movements. Blue dots are the reflected laser stimulus representation. Red arrows indicate movement trajectories. (Right) Example frames from behavior recording during grasping-like movement (top) and locomotion-like movement (bottom). (B) Reconstruction of the mean trajectories evoked by optogenetic stimulation of GRASP RFA (red), GRASP LFA (blue), TAP (green), and non-specific movement (light gray). Dark traces show the movement trajectory during the 2 s stimulus period. Light traces show the movement trajectory 1 s post stimulation. Black circle indicates the forelimb start point. Error bars represent SEM. (C) Average light-based motor maps for GRASP movement (red) and TAP movement (green). White crosses represent the maps’ center of mass and cross-bar lengths represent SEM (GRASP RC = 1.8 ± 0.2 mm; GRASP LM = 1.8 ± 0.2 mm; TAP RC = −1.0 ± 0.2 mm; TAP LM = 1.6 ± 0.2 mm; n = 8). (D) Representative average calcium responses to the optogenetic stimulus train (10 ms, 16 Hz, 2 s) at increasing laser powers. The calcium response was extracted from an ROI placed over the site of stimulation. Yellow line represents the calcium response threshold associated with complex movement execution. Blue shading represents the stimulation period. Shading indicates SEM (n_mice_ = 1; n_train_ = 3). (E) Representative wide-field image sequences of cortical activation at different laser powers. White dot indicates bregma. Red dot represents the site of stimulus. Dashed lines indicate the stimulus period. Scale bar, 1 mm. (F) (Left) Calcium transients evoked at the minimum laser power (TAP, n = 11; GRASP, n = 11). Black line indicates average calcium transient. (Right) Representative image sequences of cortical activation at minimum evoking power in two extremes (lower and higher power). Red dot represents the site of stimulus. Dashed lines indicate the stimulus period. Yellow dashed dots indicate the ROI where the calcium transients were calculated. White dot indicates bregma. Scale bar, 1mm. (G) Linear regression between power thresholds and evoked calcium transient amplitudes recorded in the same animal (TAP_intercept_ = 16.7 ± 1.8; TAP_slope_ = −0.3 ± 0.4; GRASP_intercept_ = 11.4 ± 1.7; GRASP_slope_ = 0.2 ± 0.2; n = 11).

To map forelimb multijoint movements, we performed optogenetic stimulation of several sites in the motor cortex identifying the locomotion-like movement (TAP; Video S1) and the grasping-like movement (GRASP; Video S2) (Figure 2A). To establish the movement-specific LBMMs, we initially stimulated the previously reported stereotaxic references for the RFA (+2 mm AP, +1.25 mm lateromedial [LM]) and the CFA (+0.25 mm AP, +1.5 mm LM), in order to induce the GRASP and the TAP movements, respectively (Figure 2B) (Hira et al., 2013, 2015; Tennant et al., 2011). Stimulus trains at increasing laser power were used to identify the threshold required to elicit a clear motor behavior within subjects (Figures 2D and 2E). The laser power thresholds were then employed to design the GRASP and TAP LBMMs (Figure 2C). As previously reported, we found that GRASP and TAP LBMM stereotaxic references were centered in the RFA and CFA, respectively (Harrison et al., 2012; Hira et al., 2015). The large range of power thresholds (Figure 2F, 1.3–13.2 mW) could be ascribed to both biological and ChR2 expression variability between subjects. In contrast, the same animals showing variability in the optogenetic parameters showed limited variability in the evoked calcium transient amplitude, which reflects the neuronal ensemble activation (GRASP ΔF/F_peak_ = 15.5% ± 1%; TAP ΔF/F_peak_ = 12.7% ± 1%, n = 11; Figure 2G). These observations were further confirmed by the lack of a significant relationship between the stimulus intensities and the amplitude of the evoked calcium transients (Figure 2G). Interestingly, the calcium response recorded at the site of stimulation correlated with the evoked-movement amplitude, suggesting that the calcium dynamic anticipates the movement onset (Figure S2F). Early cortical recruitment has also been reported for natural grasp movement (Quarta et al., 2020). Finally, to rule out any ceiling effects, we tested the cortical response to stimulation at a power level above the threshold required to evoke complex movements (Figures S2D and S2E). Our result shows that over-threshold stimulation increased calcium response amplitude without affecting movement kinematics, as previously reported (Harrison et al., 2012). These results suggest a critical activation threshold for triggering complex movement execution.

**Figure 3:**
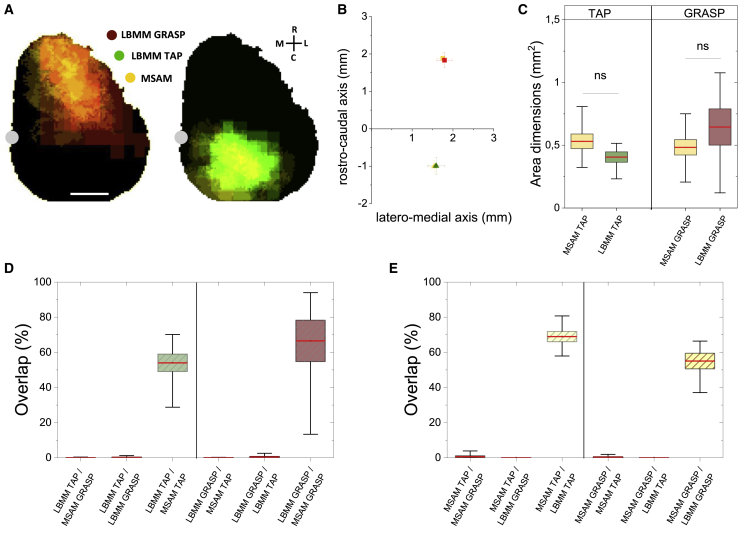
Movement-specific cortical functional connectivity is bounded to discrete modules (A) Representative schemes of cortical movement representations (left, LBMM GRASP n = 7, red; right, LBMM TAP n = 8, green) and their related average movement-specific activation map (MSAM; yellow; GRASP n = 7 and TAP n = 8). Gray dot indicates bregma. Scale bar, 1 mm. (B) Centers of mass of the LBMM (TAP MSAM, LM = 1.5 ± 0.1 mm, RC = −1.0 ± 0.2 mm; TAP LBMM, LM = 1.6 ± 0.2 mm, RC = −1.0 ± 0.2 mm; n [TAP] = 8. GRASP MSAM, LM = 1.7 ± 0.1 mm, RC = 1.9 ± 0.2 mm; GRASP LBMM, LM = 1.7 ± 0.2 mm, RC = 1.8 ± 0.2 mm, n [GRASP] = 7). Colors as in (A). Cross-bar lengths represent SEM. (C) Quantification of the area dimensions of the TAP LBMM (green), the GRASP LBMM (red), and the relative MSAMs (yellow). The red line corresponds to the mean, the box shows the standard error range, and whisker lengths are the extreme data points (TAP MSAM = 0.53 ± 0.06 mm^2^; TAP LBMM = 0.40 ± 0.04 mm^2^; n [TAP] = 8; GRASP MASM = 0.48 ± 0.06 mm^2^; GRASP LBMM = 0.64 ± 0.14 mm^2^; n [GRASP] = 7, two-tailed t test). (D and E) Comparison of the LBMM (D) and the MSAM (E) overlap (n = 7). The box shows the standard error range, and whiskers lengths are the extreme data points.

**Figure 4:**
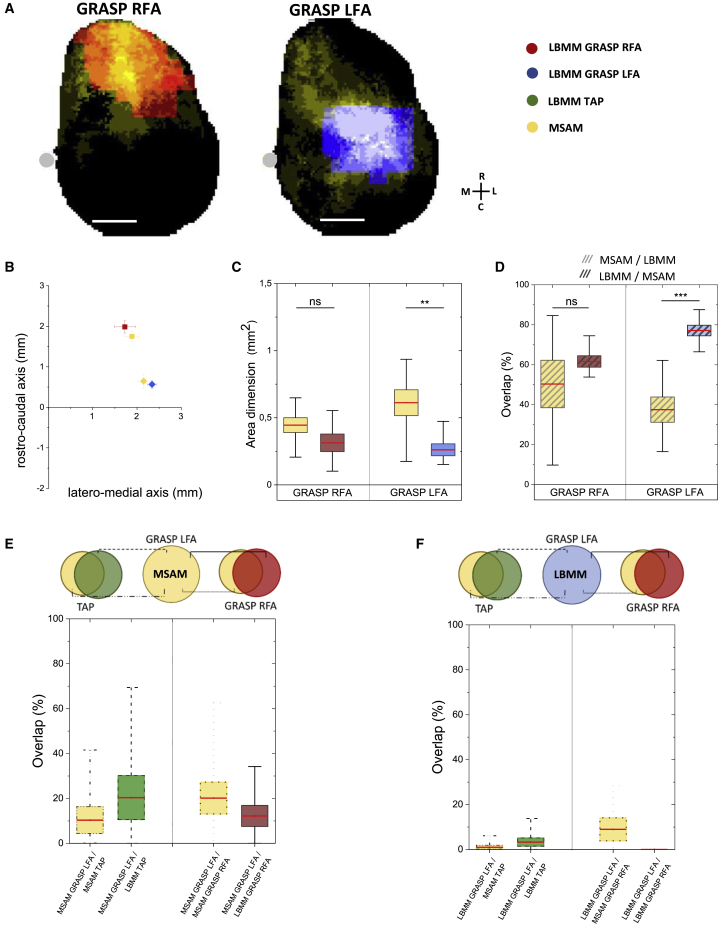
Identification of the lateral forelimb area (LFA) as a distinct grasping representation module (A) Representative schemes of the cortical movement representations (GRASP RFA in red; GRASP LFA in blue) and their related average MSAM (yellow) (n = 7). Gray dot indicates bregma. Scale bar, 1 mm. (B) Centers of mass of GRASP RFA LBMM (red), GRASP LFA LBMM (blue), and MSAMs (yellow) (LBMM RFA_RC_ = 2.0 ± 0.2 mm; RFA_LM_ = 1.7 ± 0.2 mm vs. LFA_RC_ = 0.6 ± 0.1 mm; LFA_LM_ = 2.3 ± 0.6 mm; MSAM RFA_RC_ = 1.7 ± 0.1 mm; RFA_LM_ = 1.9 ± 0.2 mm vs. LFA_RC_ = 0.6 ± 0.2 mm; LFA_LM_ = 2.1 ± 0.1 mm; n = 7). (C) Quantification of the area dimensions of the LBMMs and the relative MSAM (GRASP RFA MSAM = 0.44 ± 0.06 mm^2^; GRASP RFA LBMM = 0.31 ± 0.07 mm^2^; GRASP LFA MSAM = 0.61 ± 0.09 mm^2^; GRASP LFA LBMM = 0.26 ± 0.04 mm^2^; n = 7, ^∗∗^p < 0.01 two-tailed t test). (D) Quantification of the overlay between MSAMs and LBMMs per movement category (GRASP RFA MSAM/LBMM = 50% ± 12%; GRASP LFA MSAM/LBMM = 38% ± 6%; GRASP RFA LBMM/MSAM = 61% ± 3%; GRASP LFA LBMM/MSAM = 77% ± 2%; n = 7, ^∗∗∗^p < 0.001 two-tailed t test). Red lines indicate mean values, boxes show the standard error range, and whisker length represents the extreme data points. (E) Multiple comparison between GRASP LFA MSAM and the other movement category maps (n = 7). (F) Multiple comparison between GRASP LFA LBMM and the other movement category maps (n = 7).

**Figure 5:**
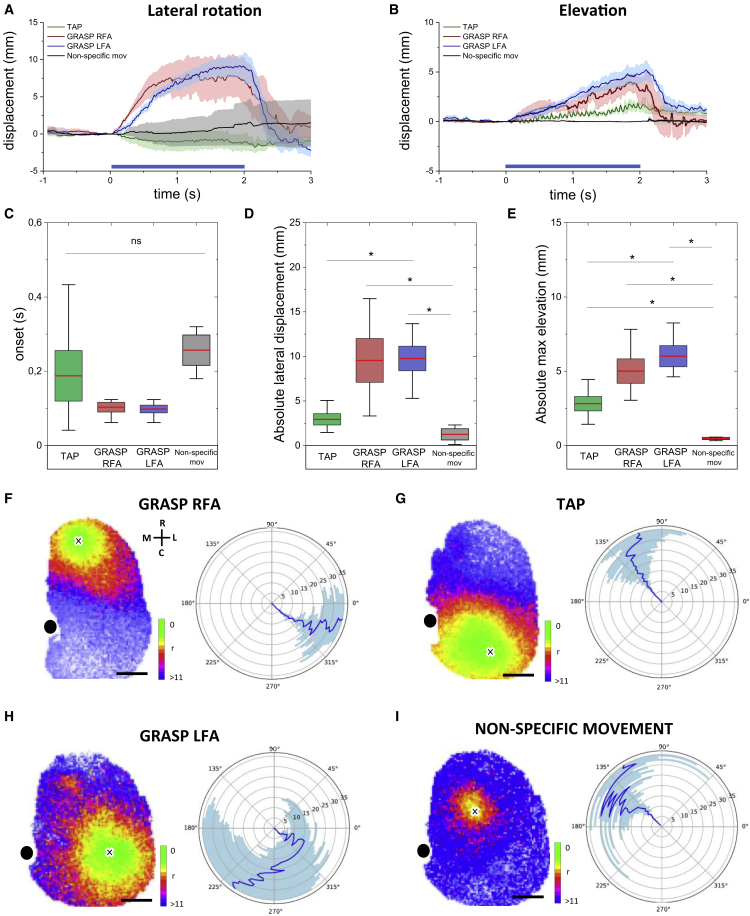
Cortical activity propagation analysis reveals movement-specific spatiotemporal patterns of activation (A) Mediolateral forelimb displacement profiles. Dark traces represent the average mediolateral displacement per movement category. Shading indicates SEM. Dark blue line at the bottom shows the stimulus period. (B) As in (A), mean elevation displacement along the y axis. (C) Box-and-whisker plots showing the onset time per movement type (TAP, 0.19 ± 0.07 s; GRASP RFA, 0.10 ± 0.01 s; GRASP LFA, 0.10 ± 0.01 s; n = 5; non-specific mov, 0.26 ± 0.04 s, n = 3; one-way ANOVA with *post hoc* Bonferroni test). The box shows the standard error range, and whiskers lengths are the extreme data points. (D) Comparison of the absolute maximum displacement along the mediolateral axis (TAP, 2.9 ± 0.6 mm; GRASP RFA, 9.5 ± 2.5 mm; GRASP LFA, 9.8 ± 1.4 mm; n = 5; non-specific mov, 1.2 ± 0.6, n = 3; ^∗^p < 0.05; one-way ANOVA with *post hoc* Bonferroni test). The box shows the standard error range, and whiskers lengths are the extreme data points. (E) Comparison of the absolute maximum elevation (TAP, 2.81 ± 0.48 mm; GRASP RFA, 5.01 ± 0.83 mm; GRASP LFA, 6.01 ± 0.71 mm; n = 5; non-specific mov, 0.47 ± 0.08 mm, n = 3; ^∗^p < 0.05; one-way ANOVA with *post hoc* Bonferroni test). The box shows the standard error range, and whiskers lengths are the extreme data points. (F) (Left) Average map of the spatiotemporal activity propagation during GRASP RFA stimulation. (Right) Polar plot, centered on the stimulation site, showing the average propagation direction. Blue line represents the radius-dependent circular mean. Shading represents the standard deviation. (G–I) As in (F), spatiotemporal activity propagation maps and polar plots are shown in (G), (H), and (I) for TAP, GRASP LFA, and non-specific movement stimulations, respectively. Scale bars, 1 mm. Color bars, pixel ranks from 0 to <11 (n_mice_ = 7; n_train_ = 20).

Video S1. TAP evoked by CFA stimulation, related to Figures 2–5 Representative video showing a frontal view of a head-fixed mouse during optogenetic stimulation of the CFA. The animal performed a locomotion-like movement by repetitively retracting and lifting the left forelimb during the laser train stimulation (flashing blue dots on the bottom left of the video).

Video S2. GRASP evoked by RFA stimulation, related to Figures 2–5 Representative video showing a frontal view of a head-fixed mouse during optogenetic stimulation of the RFA. The animal performed a grasping-like movement by closing the forepaw, turning the wrist, and moving the forelimb toward the mouth during the laser train stimulation (flashing blue dot on the bottom left of the video).

### Movement-specific cortical functional connectivity is bounded to discrete modules

We wanted to clarify the intrinsic connectivity and the possible recruitment of both RFA and CFA during the execution of the evoked movements (i.e., GRASP for RFA and TAP for CFA). To this aim, we analyzed the LBMM and the related cortical activation map for each movement category. First, we evaluated the calcium transients evoked during GRASP, TAP, and no movement (Figure S3). The results showed no significant differences between GRASP and TAP calcium transient amplitudes (Figure S3A). In addition, compared with the surrounding no-movement-evoking sites, both these areas showed a significantly higher peak amplitude (Figure S3B). This result suggests a stronger network activation in movement-evoking areas compared with sites where no macroscopic movements were stimulated. To study the spatial distribution of the activated areas, we performed maximum intensity projections (MIPs) of the imaging stacks recorded during light-based motor mapping (Figure 3A, STAR Methods), identifying the movement-specific activation map (MSAM; Figure S3). Therefore, the MSAMs allow us to identify the principal cortical areas active during a specific movement (GRASP or TAP). Subsequently, to investigate the connectivity of the engaged cortical regions, we compared the LBMM and MSAM spatial profiles (Figure 3A). The results showed that the MSAM area dimension and center of mass were comparable to the related LBMM (Figures 3B and 3C). In detail, MSAM GRASP was clustered in a module that overlapped the respective movement cortical topography (LBMM). The same result was obtained for MSAM TAP and its respective LBMM (Figure 3D). Interestingly, GRASP MSAM and TAP MSAM presented limited overlap, suggesting strong segregation of their relative activated networks (Figures 3D and 3E). These results showed that during movement stimulation there was a large cortical activation confined to the relative movement representation area.

### Identification of the lateral forelimb area as a distinct grasping representation module

Although optogenetically evoked grasping-like and locomotion-like movements were elicited by stimulating the RFA and CFA, respectively, we observed more laterally and caudally points evoking the GRASP movement that were outside the canonical border of the RFA and closer to the lateral border of the CFA (Figure 2, Figure 3C and 3A) (Tennant et al., 2011). In half of the subjects examined, these two cortical regions evoking GRASP were spatially separated by no movement-evoking points. We therefore referred to them as the RFA and the lateral forelimb area (LFA) (Figure 4A). The other half of the animals showed instead a joined RFA-LFA LBMM that was larger and laterally extended if compared with those animals who showed LFA clearly separated. This extended GRASP LBMM was then separated into two distinct areas, one frontal matching the RFA and the other matching the segregated LFA (Figure 4A, STAR Methods).

Initially, we observed that LFA- and RFA-evoked calcium transients were similar (Figure S4), evidencing a comparable local neuronal response to the optogenetic stimulus. To understand whether the LFA represented a distinct module, or whether it was an extension of the RFA network, we analyzed the LFA connectivity and its relationship with the RFA and CFA. Our results showed that, as for RFA and CFA, the LFA presented a clear matching of its LBMM and MSAM, and their centers of mass were drastically separated from the others (Figures 4B and 4D). Remarkably, the limited overlap of the GRASP LFA MSAM with the maps of the other modules reinforced the hypothesis of the discrete functional area (Figures 4E and 4F). Interestingly, we observed a modest overlap between the MSAM and the LBMM of the LFA (Figure 4D), indicating wider connectivity that exceeded the LBMM borders of the LFA reaching distant areas (Figure 4A). These results show that the LFA is associated with specific cortical connectivity features different from those of RFA or CFA.

### Grasping-like behaviors evoked in RFA and LFA exhibit similar kinematic profiles

At a glance, GRASP RFA and GRASP LFA movements presented similar profiles showing an initial forelimb displacement toward the midline followed by elevation to the mouth, which was often coupled with forepaw twisting and licking (Figure 2A; Videos S2 and S3). Therefore, to examine in detail their trajectories, we tracked the contralateral forelimb movements and performed a kinematic analysis. Although the GRASP LFA average trajectory was slightly wider compared with GRASP RFA (Figures 5A and 5B), the absolute maximum lateral displacement and elevation were similar (Figure 5D–5F). Moreover, the movement onset time did not show a significant difference between all movement categories (Figure 5C). Conversely, TAP movements displayed completely different trajectories compared with GRASP. Indeed, the locomotion-like movement was rhythmic, whereas the grasping-like was a discrete movement. TAP kinematics showed periodicity (Figure 5B), narrow medial-lateral displacement (Figure 5D), and weak elevation (Figure 5E). As expected, the stimulation of no-movement-evoking sites resulted in a remarkably reduced and non-specific forelimb displacement (Figures 5D and 5E). Overall, the similarity between GRASP RFA and GRASP LFA kinematics suggests that both cortical modules elicit the same movement.

Video S3. GRASP evoked by LFA stimulation, related to Figures 2–5 Representative video showing a frontal view of a head-fixed mouse during optogenetic stimulation of the LFA. The animal performed a grasping-like movement by closing the forepaw, turning the wrist, and moving the forelimb toward the mouth during the laser train stimulation (flashing blue dot on the bottom left of the video).

### Cortical activity propagation analysis reveals movement-specific spatiotemporal patterns of activation

The spatial analysis highlighted that the optogenetically evoked complex movements were associated with discrete modules of cortical activation. To investigate the spatiotemporal progression of this activation through cortical areas, we computed the cortical activity propagation map by ranking the time of activation of each pixel in the field of view (FOV) (Figures 5F–5I and Data S1). From a global (i.e., across the different animals) analysis of these maps, we obtained four polar plots (one for each stimulation condition) describing the spatial propagation direction. The results showed that during RFA stimulation there was a rapid activation of the area around the site of stimulus (green color, fifth rank or earlier) followed by a laterocaudal activation flow that largely preserves its spatial orientation, as shown in Figure 5F. A specular rostromedial flow of activation was observed during CFA stimulation (Figure 5G). Interestingly, LFA stimulation evoked a more complex pattern of cortical activation, and the analysis pipeline hardly provides a clear direction for the activation flow (Figure 5H). In addition, no-movement-evoking sites showed a slower, spatially limited, and almost isotropic cortical activation (Figure 5I). Overall, these results reveal that different cortical modules are linked with specific spatiotemporal patterns of activity propagation leading to complex movements. Interestingly, LFA showed propagation features that reinforce the hypothesis that the LFA relates to a specific grasping-evoking module independent of the RFA. In addition, the stimulation of movement-related cortical areas leads to a more marked flow of activation compared with no-movement-evoking areas, suggesting the persistence of more complex connectivity associated with movement execution.

### Excitatory synaptic block leads to movement impairment and disruption of the associated connectivity features

**Figure 6:**
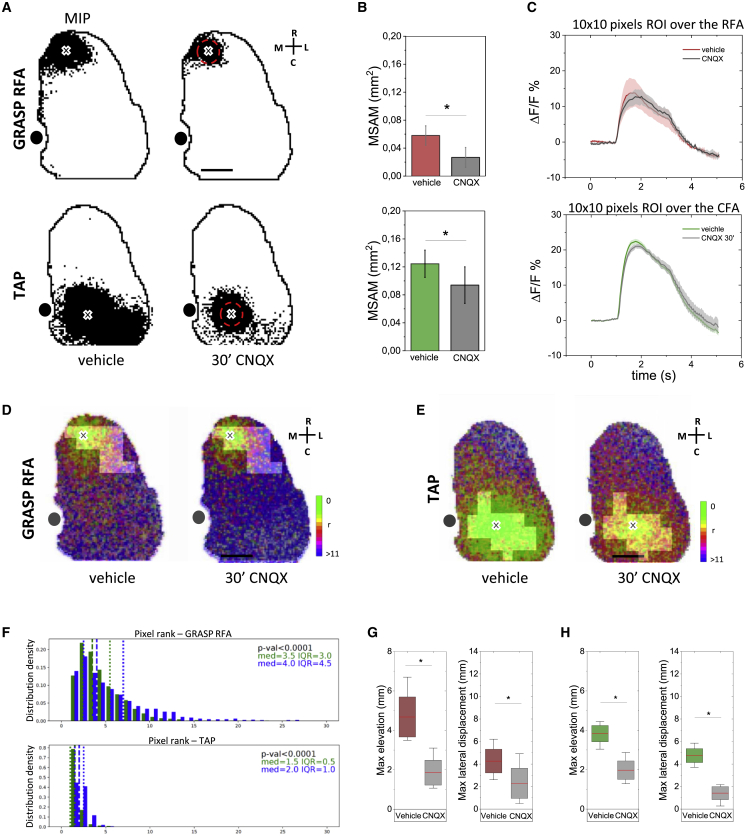
Excitatory synaptic block leads to movement impairment and disruption of the associated connectivity features (A) Representative MIP showing optogenetically evoked cortical activation during vehicle (left) and CNQX topical application (right) in RFA (top) and CFA (bottom). Cross represents the stimulus site. Red dashed lines indicate the CNQX topical application site. Black dot represents bregma. Scale bar, 1 mm. (B) Quantification of the effect of CNQX topical application on MSAM extension in RFA (top) and CFA (bottom) (GRASP, vehicle = 0.05 ± 0.01 mm^2^ vs. CNQX = 0.02 ± 0.01 mm^2^, n = 3; TAP, vehicle = 0.12 ± 0.02 mm^2^ vs. CNQX = 0.09 ± 0.02 mm^2^, n = 3; ^∗^p < 0.05, paired-sample t test). (C–E) Averaged evoked calcium transient profiles (C), recorded from an ROI placed over the site of stimulation, in vehicle and following CNQX topical application in RFA (top) and CFA (bottom) (GRASP, vehicle 14.72 ± 4.20 ΔF/F vs. CNQX 11.62 ± 3.66 ΔF/F, n = 3, paired-sample t test; TAP, vehicle 25.80 ± 1.31 ΔF/F vs. CNQX 21.26 ± 0.63 ΔF/F, n = 3, paired-sample t test). Shading represents SEM. Representative activity propagation maps of GRASP RFA (D) and TAP (E) showing the effects of CNQX topical application. Bright areas represent the LBMM. Scale bar, 1 mm. Color bar, pixel ranks from 0 to <11. (F) Pixel rank distribution of the region corresponding to the LBMM (bright area in [D] and [E]) for GRASP RFA (top) and TAP (bottom), before (green) and after (blue) CNQX topical application (n = 3). med, median; IQR, interquartile range; Wilcoxon signed-rank test. (G) CNQX topical application effect on GRASP RFA kinematics. Comparison of the absolute left-forelimb maximum elevation (left) and the absolute maximum displacement along the mediolateral axis (right) in vehicle (brown) and CNQX topical application (gray) in RFA (maximum elevation, vehicle 4.6 ± 1 mm vs. CNQX 1.8 ± 0.6 mm; GRASP maximum lateral displacement, vehicle 4.2 ± 1 mm vs. CNQX 2.2 ± 1 mm, n = 3; ^∗^p < 0.05, paired-sample t test). Red lines indicate means, boxes show the standard error range, whisker length represents the extreme data points. (H) CNQX topical application effect on TAP CFA kinematics. Comparison of the absolute left-forelimb maximum elevation (left) and the absolute maximum displacement along the mediolateral axis (right) in vehicle (green) and CNQX topical application (gray) in CFA (maximum elevation, vehicle 3.8 ± 0.4 mm vs. CNQX 1.9 ± 0.4 mm; maximum lateral displacement: vehicle 4.7 ± 1 mm vs. CNQX 1.4 ± 1 mm, n = 3; ^∗^p < 0.05, paired-sample t test). Red lines indicate means, boxes show the standard error range, and whisker length represents the extreme data points.

The spatiotemporal analysis revealed a progressive engagement of specific regions in the motor cortex. A common strategy to dissect the role of different functional nodes in a neuronal network is the pharmacological synaptic transmission block, in particular, using glutamatergic transmission antagonists (Kuhn et al., 2008; Minlebaev et al., 2007). To investigate both the role of the local connectivity and the reciprocal role of each module during optogenetically evoked movement execution, we performed a module-specific block of the excitatory synaptic transmission through topical application of the AMPA/kainate receptor antagonist 6-cyano-7-nitroquinoxaline-2,3-dione (CNQX) on the cortical surface (Harrison et al., 2012; Kuhn et al., 2008; Spalletti et al., 2017). To ensure an extended inhibition, we made an ∼1/1.5-mm-diameter cranial window covering almost all the selected LBMM areas (GRASP RFA 0.31 ± 0.06 mm^2^; TAP 0.40 ± 0.04 mm^2^). The results showed that CNQX application in RFA reduces the extension of the GRASP RFA activation map (Figure 6B), while it does not significantly affect the calcium transient profiles (Figure 6B). Similar results were obtained by applying CNQX in CFA (Figures 6B and 6C). These results suggest an impairment of the local connectivity (a decrease in activation spreading) that does not affect the direct local response to the optogenetic stimulus. These findings are in accordance with evidence showing that topical application of CNQX disrupts cortical connectivity while preserving the direct activation of ChR2-expressing neurons (Harrison et al., 2012). Moreover, to analyze the effect of the module-specific block of the excitatory synaptic transmission on the activity propagation features, we compared the pixel rank distribution of a region of interest (ROI) overlapping the relative LBMM before and after the pharmacological block (Figures 6D and 6E). These results highlight an increase in both the median and the interquartile range (IQR) caused by the pharmacological connectivity interference, suggesting a slower and more disorganized propagation of the cortical activity, respectively (Figure 6F). It should be noted that these results correlate with the behavioral outcome (Figure 6G). Indeed, as previously reported by Harrison and colleagues, CNQX application leads to faults and distortions in complex movement execution, until the complete extinction of a recognizable complex movement (Harrison et al., 2012). Interestingly, CNQX application in RFA resulted in a block of GRASP execution while preserving the CFA-evoked TAP movement. The same result was obtained following the application of CNQX in CFA that resulted in a block of the TAP while maintaining a successful GRASP RFA expression. Taken together, these results suggest that local excitatory synaptic inputs could be required to control complex motor behaviors and that GRASP RFA and TAP could be controlled by two independent cortical modules.

### LFA-evoked grasping does not require RFA activation

**Figure 7:**
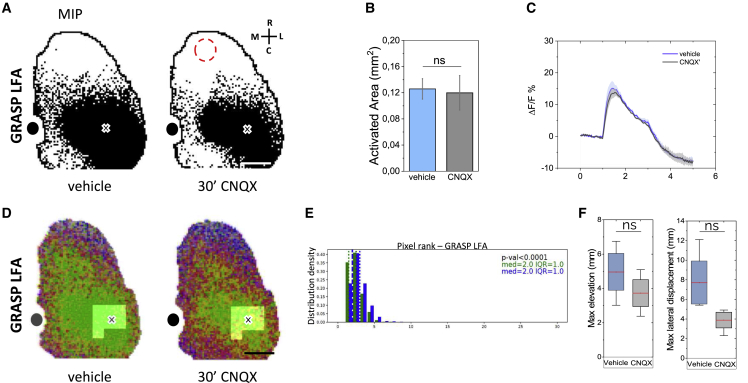
LFA-evoked grasping does not require RFA activation (A) Representative MIPs showing optogenetically evoked cortical activation in LFA during RFA topical application of vehicle (left) and CNQX (right). Cross represents the stimulus site. Red dashed line indicates the CNQX topical application site. Black dot represents bregma. Scale bar, 1 mm. (B) Quantification of the effect of CNQX topical application in RFA on LFA MSAM extension (vehicle 0.12 ± 0.01 mm^2^ vs. CNQX 0.11 ± 0.02 mm^2^; n = 3; paired-sample t test). (C) Averaged LFA evoked calcium transient profiles in vehicle and following CNQX topical application in RFA (vehicle 15 ± 2 ΔF/F vs. CNQX 13 ± 1 ΔF/F; n = 3; paired-sample t test). Shading represents SEM. (D) Representative activity propagation maps of GRASP LFA showing the effect of CNQX topical application in RFA. (E) Pixel rank distribution of the region corresponding to the LBMM (bright area in [D]) for GRASP LFA, before (vehicle) and after CNQX topical application (n = 3). med, median; IQR, interquartile range; Wilcoxon signed-rank test. (F) Effects of RFA CNQX topical application on GRASP LFA kinematics. Comparison of the absolute left forelimb maximum elevation (left) and the maximum lateral displacement (right) following LFA stimulation in vehicle and RFA CNQX topical application (maximum elevation, vehicle 4.9 ± 1.8 mm vs. CNQX 3.7 ± 1.4 mm; lateral displacement, vehicle 7.7 ± 2.0 mm vs. CNQX 1.3 ± 1.0 mm; n = 3; paired-sample t test). Red lines indicate means, boxes show the standard error range, and whisker length represents the extreme data points.

Connectivity analysis showed that the three identified functional modules activate distinct cortical regions during movement execution, and the excitatory synaptic block experiments suggested that GRASP RFA and TAP do not require mutual activation (Figure 6). To further study the relation between LFA and RFA, we stimulated the LFA during RFA pharmacological block (Figure 7). The findings revealed that neither the LFA MSAM dimension (Figure 7B) nor the evoked-calcium transients (Figure 7C) had significant differences. Moreover, we observed that the RFA pharmacological block did not modify the LFA spatiotemporal propagation features (Figures 7D and 7E). Interestingly, the preserved cortical connectivity of the LFA correlated with the successful execution of the GRASP LFA movement despite the block in RFA (Figure 7F). These results suggest that the LFA motor output does not require the RFA activation.

## Discussion

### Large-scale all-optical manipulation and readout of cortical dynamics

To causally investigate neuronal circuits, optogenetics has been paired with single- and two-photon fluorescence imaging in all-optical neurophysiology approaches (Chen et al., 2018; Emiliani et al., 2015). There are still important limitations in matching optogenetics and fluorescence imaging due to the large cross talk between functional indicators and optogenetic actuators, caused by their spectral overlap. This phenomenon leads to spurious modulation of neuronal activity during imaging or stimulation artifacts in the readout channel, making it hard to develop a cross-talk-free all-optical method (Ronzitti et al., 2018; Soor et al., 2019). Recently, Forli and colleagues took advantage of two-photon absorption for both jRCaMP1a excitation and ChR2 activation to manipulate and record cortical neuronal activity in anesthetized mice, demonstrating this solution as the best option to drastically reduce cross talk (Forli et al., 2018). Here we extended this configuration to transcranial single-photon excitation in awake head-fixed mice. We initially evaluated the cross-activation of jRCaMP1a and ChR2 using visible-light excitation. The electrophysiological analysis showed that the imaging excitation did not affect the LFP content, suggesting that there is no detectable neuronal activation during jRCaMP1a imaging (Figures S1A and S1B). This result is in line with previous evidence showing the absence of cross talk using RCaMPs and ChR2 (Akerboom et al., 2013; Dana et al., 2016; Forli et al., 2018). Using the patch-clamp technique, it has been demonstrated that single-photon wide-field illumination at 590 nm of cultured neurons expressing ChR2 prevents photocurrent generation, thus maintaining subthreshold potentials (Forli et al., 2018). The second aspect we considered was the jRCaMP1a activation following blue-laser excitation. As shown in Figure S1C the laser stimulation did not change the jRCaMP1a fluorescence dynamics in mice lacking ChR2, demonstrating that optogenetic stimulation did not affect the neuronal activity readout. In addition, we optimized a transfection strategy to achieve a wide and stable expression of both the optogenetic actuator and the fluorescence reporter over one hemisphere, exploiting a double AAV vector injection. Consistent with our previous study (Montagni et al., 2019), we obtained stable expression for both jRCaMP1a and ChR2 in the right hemisphere motor cortex (Figure 1B).

### Segregated functional organization of mouse forelimb representations

Due to technical limitations, investigations of the cortical connectivity related to natural behaviors (Makino et al., 2017; Quarta et al., 2020) and motor mapping studies (Guo et al., 2014) are largely confined to separated experiments. Previous research showed that a rich repertoire of complex movements can be evoked by optogenetically stimulating different sites in the mouse motor cortex (Harrison et al., 2012; Hira et al., 2015). In the present study, we developed a method to analyze the cortical representation of complex movements and their related activation features. We focused on two forelimb movements, the discrete forepaw-to-mouth movement (GRASP) and the rhythmic locomotion-like movement (TAP) obtained by stimulating the RFA and CFA, respectively(Ayling et al., 2009; Hira et al., 2009, 2015). According to the literature, the quantitative kinematic analysis revealed that the TAP trajectory exhibited rhythmic repetitions and a slight lateral displacement, while the GRASP trajectory was mainly displaced in the mediolateral plane and exhibited a discrete elevation of the forelimb toward the mouth (Figure 5) (Harrison et al., 2012). Nevertheless, the observed movements were characterized by larger onset times compared with the literature; this discrepancy could be ascribed to both the lower stimulation frequency and the different animal models used.

To map the cortical topography of these movements, we evaluated the minimum laser power required to elicit a clear GRASP or TAP by gradually increasing the stimulus intensity. The results showed variable subject-specific power threshold values, whereas the related cortical activation exhibited a slight between-subjects heterogeneity (Figure 2). This difference could be ascribed to the between-subjects variability. Indeed, the minimum stimulation intensity to evoke a movement (i.e., power threshold) is subject specific; therefore, a range of stimulation intensities is always used in studies employing electrical (Bonazzi et al., 2013; Graziano et al., 2002) or optogenetic stimulation (Ayling et al., 2009; Hira et al., 2015). Other sources of variability are the transfection efficiency, which is the number of neurons expressing the ChR2; the level of transfection; and the number of ChR2 copies expressed by a single neuron. Interestingly, although transcranial optogenetic stimulation of movements was first developed in Thy1-ChR2 transgenic mice, a mouse line that stably expressed ChR2 in excitatory neurons, a range of laser power was used to evoke the same movement in different animals. It should be noted that the range of power used in these works was very close to ours (0.5–10 mW), although a more stable ChR2-expressing animal model was used (AAV transfection vs. transgenic mice) (Harrison et al., 2012). Once the subject-specific minimum laser power was identified, we designed the GRASP and TAP LBMMs. In accordance with previous studies, we found that the GRASP and TAP LBMMs covered the RFA and CFA, respectively (Figure 2C) (Harrison et al., 2012; Hira et al., 2015). Our all-optical tool allowed us to study the amplitudes of the calcium transients evoked in GRASP and TAP LBMMs, which were significantly different from those evoked in no-movement-evoking areas (Figure S2C). This result suggests stronger intrinsic connectivity of the complex movement representation areas. Moreover, our spatial analysis revealed that the GRASP representation extended laterally beyond the RFA toward the forelimb somatosensory (FLS1) cortex (Figures 2C and 3A). Therefore, we characterized this lateralization, identifying the LFA (Figure 4). The fact that in half of the animals the motor maps showed a jointed RFA-LFA could be ascribed to the intersubject variability. Indeed, the mouse motor map can change profoundly between subjects (Tennant et al., 2011). Moreover, previous works demonstrated that the spatial resolution of light-based mapping is strongly affected by the stimulus intensity (Ayling et al., 2009; Harrison et al., 2012; Tandon et al., 2008). Therefore, it is reasonable that in some animals we observed slight differences in the segmentation of the motor areas, as RFA and LFA stimulation evoked similar movements, making their separation difficult without exploiting the MSAMs.

Interestingly, Bonazzi and colleagues mapped the motor cortex topography in anesthetized rats through intracranial microstimulation (ICMS), describing a lateral area expressing a hold-like forelimb movement, defined as the paw supination (the wrist and forearm turning toward the midline or the face) (Bonazzi et al., 2013). In the lateral part of the rat motor map, the authors showed that the hold-like movement is coupled with elevation and abduction movements that resemble the feature considered for grasping movement. Moreover, Harrison and colleagues, by exploiting the light-based motor mapping technique, observed a lateral extension of the forelimb abduction movement centered in the RFA (Harrison et al., 2012). Previous studies reported also that corticospinal motor neurons (CSNs) can be found in RFA, CFA, and a small, circumscribed, cluster in the secondary somatosensory cortex named PL-CFA (Wang et al., 2017; Wise et al., 1979). At the spinal cord level, CSN axons from PL-CFA mainly overlap with the RFA-CSN premotor neurons (Suter and Shepherd, 2015; Wang et al., 2017), suggesting the control of the same group of muscles. Therefore, the LFA that we functionally characterized in this paper could anatomically refer to the group of neurons described in these previous works. In our hypothesis, the fact that half of the animals had clearly segregated RFA and LFA regions could be ascribed to (1) the small separation between them, (2) the interanimal variability in the motor representations (extension and localization), and (3) the variability in the minimum power to evoke movements. Indeed, it is possible that in some animals the minimum laser power to evoke a movement was sufficient to slightly activate the LFA when stimulation was targeted on the border of the RFA. In these cases, the resulting motor map did not show a clear separation between the RFA and the LFA. This is in line with what was previously reported by Harrison and colleagues, that the spatial resolution of the motor maps is affected by light scattering and the activation spreading. Interestingly, the authors reported either unified or segregated grasping maps (Harrison et al., 2012).

Optogenetic stimulation of RFA and LFA led to the generation of similar grasping behaviors; therefore, we investigated the functional role of the LFA compared with the RFA to test the hypothesis of two distinct grasping representation areas. The kinematic analysis results showed that GRASP RFA and GRASP LFA expressed comparable trajectories achieving equal elevation and displacement in the medial-lateral plane and displaying the same onset time (Figures 5F–5I). This result confirms that LFA and RFA exhibit similar motor outputs.

To study the connectivity hallmarks of GRASP RFA, GRASP LFA, and TAP, we calculated the MSAMs. We found that the MSAMs were packed in segregated modules that largely overlapped the associated movement representation topography (Figures 3 and 4). Indeed, the GRASP RFA topography and MSAM circumvent the TAP area and the relative MSAM. The same results were observed for the TAP movement. Remarkably, also, the LFA activation map avoided the RFA- and CFA-relative LBMMs and MSAMs, displaying specific connectivity features (Figure 4). We wondered if any network effects stemming from peripheral feedback could affect the MSAMs. Interestingly, Harrison and colleagues previously investigated cortical activity related to feedback from the periphery by imaging the intrinsic optical signal mapping the somatosensory cortical representation of the forelimb (sFL) (Harrison et al., 2012). The authors showed that the sFL only partially overlaps the more lateral part of the RFA. In agreement with their results, if there were any network effects stemming from peripheral feedback, sFL activity should be recruited for both GRASP and TAP MSAMs. Instead, our results show that when TAP is evoked by CFA stimulation, there is no clear sFL activation, as highlighted in the activity map (Figures 2F and S3A) and propagation analysis (Figure 5G). However, we cannot completely exclude a low level of sFL activation cut out by the threshold used to create the MASMs.

ICMS stimulation has been recently coupled with intrinsic signal optical imaging to reconstruct activation maps related to stimulated forelimb movements in squirrel monkeys (Card and Gharbawie, 2020). The authors demonstrated that the intrinsic motor cortex connectivity matched the forelimb somatotopic representation in the primary motor cortex (Card and Gharbawie, 2020). Our results are in line with evidence suggesting a segregated functional organization of CFA and RFA (Brown and Teskey, 2014; Hira et al., 2015), despite their mutual connections that may have a role in coordinating sequences of complex movements such as the reach-to-grasp behavior (Bansal et al., 2012; Stark et al., 2007). Interestingly, the spatial clustering of functionally correlated units in the motor cortex seems to be expressed across scales, from neurons (Dombeck et al., 2009) to entire functional areas (Hira et al., 2015).

Spatiotemporal activity propagation features are pivotal aspects of the computation and communication between subsystems of the brain (Riehle et al., 2013). In the motor cortex, behaviorally relevant propagating patterns of cortical activation have been demonstrated to be necessary for movement initiation (Balasubramanian et al., 2020; Takahashi et al., 2015). Therefore, we explored the spatiotemporal spreading of the neuronal activity during stimulated motor performances and we found movement-specific propagation patterns for the three motor regions (Figures 5F–5I). Our analysis revealed movement-specific orientation of the activity propagation, showing opposite directions for RFA and CFA. Conversely, LFA patterns exhibited more complex features, rather than the fairly linear propagation observed for the other modules. Indeed, the LFA activation involves distal areas that are activated early, reflecting a distributed connectivity that generates variability in the trajectory analysis. These results reinforce the idea that LFA could represent a distinct GRASP representation.

To test the correlation between the stimulated movements and the activity features observed, we performed module-specific inhibition of the excitatory synaptic transmission. This pharmacological tool allows an effective direct optogenetic stimulation of the targeted area while blocking its input connections, thus probing the role of the module-specific network in generating the forelimb movement. It has been demonstrated that topical application of the AMPA/kainate receptor antagonist CNQX on the cortical surface disrupts the optogenetically evoked complex movement execution while preserving the direct activation of ChR2-expressing neurons (Harrison et al., 2012). Accordingly, our results show that RFA pharmacological inactivation interferes with the GRASP execution while retaining the ability to evoke the TAP movement, and the specular phenomenon was observed during CFA inhibition (Figures 6 and S5), supporting the idea of two functionally independent modules (Harrison et al., 2012; Hira et al., 2015). Moreover, we reported that CNQX application leads to a significant reduction in the MSAM extension associated with slower and more disorganized patterns of local propagation (Figures 6 and S5), highlighting that the activation features we described reflect the module-specific network activity linked to movement execution.

These results sharpen the idea that direct activation of corticospinal projections is not sufficient to drive full movement performance, thus confirming the pivotal role of the cortical synaptic inputs. Further studies will be necessary to understand the contribution of the recurrent corticocortical circuits (Anderson et al., 2010; Hooks et al., 2011) or subcortical loops (Kelly and Strick, 2003) in complex movement control and to define whether the modules found in the motor cortex and their related inputs can be organized as central pattern generator networks, in which the activation of a group of neurons can be sufficient to elicit an entire motor engram (Grillner and El Manira, 2020; Sauerbrei et al., 2020). Moreover, our results suggest that during RFA inactivation the LFA behavioral output and all its activation features were preserved (Figures 7 and S5) and that the GRASP LFA expression is not affected by the RFA network.

The experimental paradigm we developed represents a powerful approach to causally dissect cortical connectivity, reaching its full potential in experimental settings where it is not possible to record behavioral outputs, for instance, in the study of non-motor cortical regions or the investigation of different brain states and pathologies in altered levels of consciousness, i.e., sleep, anesthesia, or coma (Sarasso et al., 2015). Exploiting this method, we raised evidence for a segregated functional organization of CFA and RFA and we identified a second grasping representation area, functionally independent of the RFA and expressing distinct activation features, which we named the lateral forelimb area. This previously unreachable information on the cortical circuitry could considerably help to develop a unique model of the mouse motor cortex.

### Limitations of the study

In this study, forelimb movement was recorded with a single behavioral camera that was sufficient to distinguish GRASP and TAP movements. Conversely, we are not able to distinguish fine kinematic differences that could exist between GRASP RFA and GRASP LFA. In addition, as previously reported, transcranial optogenetic stimulation has restricted control of the depth of stimulation due to the reduced light penetration depth and scattering effect in the tissue, which prevent the causal dissection of network dynamics related to different layers.

Another limitation of this study concerns the possibility of quantifying the contribution of a specific cortical layer to the activity detected. Indeed, wide-field fluorescence microscopy lacks optical sectioning, and the viral transfection labels neurons throughout the entire cortex. Thus, the recorded fluorescence signal could be considered a convolution of the contribution of different cortical layers. The use of both Cre mouse lines and targeted viral promoters to further restrict the neuronal population investigated could represent effective strategies to overcome the issues related to layer specificity.

## STAR★Methods

### Key resources table

**Table:** 

REAGENT or RESOURCE	SOURCE	IDENTIFIER
**Antibodies**		

Anti-NeuN (rabbit polyclonal)	Sigma	Cat#634301
Goat anti-Rabbit Secondary Antibody, Alexa Fluor™ 514	ThermoFisher	Cat#A-31558; RRID: AB_2536173

**Bacterial and virus strains**		

pGP-AAV9-syn-NES-jRCaMP1a-WPRE.211.1488	CliniSciences	Cat#CUST-VIR-21022018-1a
pAAV-CamKIIa-ChR2(H134R)-Cerulean	CliniSciences	N/A

**Experimental models: Organisms/strains**		

Mouse: C57BL/6Ncr	Charles River	strain #027

**Software and algorithms**		

OriginPro	OriginLab	https://www.originlab.com
ImageJ	Fiji	https://imagej.net
AnimalTracker	AnimalTracker	http://animaltracker.elte.hu/main
Phyton	Phyton	https://www.python.org/

### Resource availability

#### Lead contact

Further information and requests for resources and reagents should be directed to and will be fulfilled by the lead contact, Anna Letizia Allegra Mascaro (allegra@lens.unifi.it).

#### Materials availability

This study did not generate new unique reagents.

### Experimental model and subject details

All experiments were performed in accordance with the guidelines of the Italian Minister of Health (aut. n. 871/2018). The mice were obtained from the Charles River. The study used C57BL/6J adult mice (6–12 months) of both sexes. Mice were housed in a temperature- and humidity-controlled room, with food and water ad libitum.

### Method details

#### Virus injection and intact-skull window

C57BL/6J adult mice (6–12 months) of both sexes were anesthetized with isoflurane (3% for induction, 1–2% for maintenance) and placed in a stereotaxic apparatus (KOPF, model 1900). Ophthalmic gel (Lacrilube) was applied to prevent eye drying, body temperature was maintained at 36°C using a heating pad and lidocaine 2% was used as local anesthetic. The skin and the periosteum were cleaned and removed. bregma was signed with a black fine-tip pen. To achieve widespread expression of both jRCaMP1a and ChR2 over the right hemisphere, small holes were drilled at two coordinates (AP +2.0 mm, ML +1.7 mm; AP -0.5 mm, LM +1.7 mm from bregma). A 500 nL volume of mixed viruses (pGP-AAV9-syn-NES-jRCaMP1a-WPRE.211.1488 and pAAV9-CamKII-hChR2(H134R)-Cerulean, 1 × 10^13^ GC ml^−1^, CliniSciences, 250 nL respectively) was pressure-injected through a pulled glass micropipette at one depth per site (−0.5 mm ventral from dura surface) using an electrically gated pressure injector (Picospritzer III—Science Products, n 3 Hz, ON 4 ms) for a total volume of 1 μL per mouse. A custom-made aluminum head-bar placed behind lambda and a cover glass implanted on the exposed skull were fixed using transparent dental cement (Super Bond C&B – Sun Medical). After the surgery, mice were recovered in a temperature- and humidity-controlled room, with food and water ad libitum for two weeks before recordings.

#### Wide-field microscopy setup

Wide-field imaging and optogenetic stimulation were performed using a custom-made microscope with two excitation sources to simultaneously excite the opsin (ChR2-cerulean) and the calcium indicator (jRCaMP1a) (Conti et al., 2019). The excitation source for jRCaMP1a was a red-light beam of emitting diodes (595nm LED light, M595L3 Thorlabs, New Jersey, United State) and the excitation band was selected by a bandpass filter (578/21 nm, Semrock, Rochester, New York, USA). The light beam was deflected by a dichroic mirror (606nm, Semrock, Rochester, New York, USA) to the objective (2.5x EC Plan Neofluoar, NA 0.085) toward the skull. The excitation source for single-photon stimulation of ChR2 was a continuous wavelength (CW) laser (λ = 473 nm, OBIS 473 nm LX 75mW, Coherent, Santa Clara, CA, USA). The excitation beam was overlaid on the imaging pathway using a second dichroic beam splitter (FF484-Fdi01-25 ×36, Semrock, Rochester, New York, NY, USA) before the objective. The system has a random-access scanning head with two orthogonally-mounted acousto-optical deflectors (DTSXY400, AA Opto-Electronic, Orsay France). The laser beam nominal size was ∼200 μm. The jRCaMP1a fluorescence signal emitted was collected through a band-pass filter (630/69, Semrock, Rochester, New York, USA) and focused by a tube lens (500 nm) on the sensor of a demagnified (20X objective, LD Plan Neofluar, 20×/0.4 M27, Carl Zeiss Microscopy, Oberkochen, Germany) high speed complementary metal-oxide semiconductor (CMOS) camera (Orca Flash 4.0 Hamamatsu Photonics, NJ, USA). The camera acquired images at a resolution of 100 by 100 pixels covering a quadratic field-of-view of 5.2 by 5.2 mm^2^ of the cortex.

#### Wide-field imaging in awake mice

14 days after the injection, head-fixed imaging sessions were performed for three consecutive weeks. An animal-specific field of view (FOV) template was used to manually adjust the imaging field daily. Each imaging session consisted of 5–10 s of recording in resting-state followed by the stimulus train (2 s) and 30 s of imaging after the stimulus (sampling rate: 50 Hz). The waiting time for consecutive sessions was 3 min per animal. LED light intensity was 4 mW after the objective.

#### Transcranial optogenetic stimulation

Laser stimulation patterns were generated using two orthogonally-mounted acousto-optical deflectors controlled by a custom-written LabView 2013 software (National Instruments). A reference image of the FOV was used to target the laser beam on a selected cortex area.

*Single-pulse laser stimulation* consisted of one pulse (10 ms ON) repeated 8 times in one imaging session at different laser power (0.22–1.3 – 2.5–5.2 – 7.7–13.2 mW, after the objective).

*The stimulus train* consisted of 2 s, 16 Hz, 10 ms ON. The 16 Hz frequency resulted from a trade-off between the imaging recording frequency (50Hz) and the stimulation frequency needed to evoke a complex movement for saving at least two imaging frames during the stimulus train. For laser power calibration experiments the laser power used were: 1.3- 2.5 - 5.2–7.7 - 13.2 mW. For light-based motor mapping, connectivity studies and pharmacological inhibition laser power was the minimum power required to evoke movements (from 1.3 mW to 13.2 mW).

#### Light-based motor map (LBMM)

The LBMMs for locomotion-like (TAP) and grasping-like (GRASP) movements were obtained in separate experiments. A virtual grid (14 x 14, 364 μm spacing) was superimposed on the animal-specific FOV template using Fiji (Schindelin et al., 2012). A stimulus train was then delivered once for all points of the grid in the FOV. The stimulation site for each session was randomly selected. The left forepaw position during imaging sessions was monitored using a camera equipped with a red illumination light focused on the forepaw and not interfering with imaging. Forelimb movements were evaluated by two different expert observers and visually categorized as (i) grasping-like movements: contralateral forepaw was closed, the wrist turned and moved toward the mouth (ii) locomotion-like movements: contralateral forelimb was retracted and lifted at least twice, simulating a walking movement (iii) no-movements and movement interference: the absence of at least one movement criterion during the stimulus. The average LBMM was created by aligning three points in the FOV (bregma and injection sites) in 7 animals per movement category.

*Optogenetic of the Lateral Forelimb Area (LFA) LBMM*. 4 out of 8 animals presented segregated LFA and RFA light-based motor map. The average of these LFA LBMM, with a 100% threshold (total overlap for high restriction), was used as a mask for identifying the LFA border in those animals that presented a unified light-based motor map.

#### Calcium data analysis

In vivo *quantification of jRCaMP1a and ChR2 spatial distribution*. The full width at half-maximum (FHWM) of spatial fluorescence profile *in vivo* was evaluated during the third and fourth weeks after injection. FWHM was calculated on the average of the three brightest frames acquired in the resting state imaging session, over two parallel lines that crossed the injection sites in the mediolateral plane.

*Single-pulse correlation* was performed during the third and fourth weeks after injection. Single-pulse laser stimulations were delivered to the cortex region with the maximum level of ChR2 and jRCaMP1a expression. The consequently evoked calcium response dynamics (time series) were extracted from a region of interest (ROI, area 0,05 mm^2^) placed over the stimulation site.

*Power calibration***.** The optogenetic stimulus was delivered in the center of the light-based motor map. The stimulus train was repeated 3 times at increasing laser power to select the minimum power required to evoke GRASP and TAP movements. Calcium dynamics (time series) were extracted from a ROI (area 0.05 mm^2^) placed over the site of stimulation.

*Movement specific activation map (MSAM).* An imaging stack was recorded for each site of the grid stimulated during the light-based motor mapping. For each acquisition (14 x 14, 364 μm spacing), the Maximum-Intensity Projection (MIP) was obtained and subsequently grouped by evoked-movement category in (i) GRASP RFA movement, (ii) GRASP LFA movement, (iii) TAP movement. Since light-based motor maps for TAP and GRASP were obtained in separate experiments, there were two different groups of no-movement: (iv) GRASP-related no-movement and (v) TAP-related no-movement. An average maximum activity value based on all MIPs was then calculated for GRASP RFA/LFA and TAP categories, and half of that value has been used for thresholding the MIPs of all groups. Thresholded MIPs were averaged over single-point stimulations within the related LBMM and an additional threshold of 2x standard deviation (SD) was applied, obtaining the average activation map for all five experimental groups. Finally, in order to obtain movement-specific activation maps (MSAMs), the non-specific movement average activation maps were spatially subtracted from those related to GRASP RFA, GRASP LFA and TAP. The spatial overlap between the MSAM and the related LBMM was then assessed and quantified as a percentage of the total dimension of both the maps involved. Although our approach leads to a slightly variable expression of jRCaMP1a, all the analyses are performed following ΔF/F normalization which overcomes a possible issue related to viral expression boundaries.

#### Video tracking analysis

A machine vision camera (PointGrey flir Chamaleon3, CM3-U3-13Y3C-CS) was orthogonally set 100 mm in front of the mouse to evaluate the left forelimb movement induced by contralateral optogenetic stimulation (frame rate 100 Hz). The camera acquired images at a resolution of 800 by 600 pixels covering a field-of-view of 24 by 18 mm^2^ of the cortex (0.03 mm per pixel). Visible illumination light at 630 nm was focused on the left forepaw, to avoid imaging interference. Five individual trains per movement category for each animal were filmed (n_mice_ = 5; n_trains_ = 5). Videos were analyzed using the ImageJ plugin AnimalTracker, obtaining XY coordinates of the forelimb in each frame from a starting point (Ref.(Gulyas et al., 2016) for details). To compare evoked complex movements, we analyzed the tracked forelimb mediolateral displacement, the elevation and the speed (mm/s) for each train.

#### In-vivo local field potential recording

Local field potentials (LFPs) were recorded in the center of the transfected area. Glass pipettes were used to avoid light-induced artifacts during the electrophysiological recordings and were filled with a 2 M NaCl solution. The electrode was advanced, through a little hole in the skull, into the motor cortex L5 (800 μm from the dura surface) using a motorized micromanipulator (EXFO Burleigh PCS6000 Motorized Manipulator). Signals were amplified with a 3000 AC/DC differential amplifier, sampled at 10 kHz, highpass filtered at 0.1 Hz and lowpass filtered at 3 kHz. A reference and ground screws were placed on the occipital bone. LFP signal was recorded during a randomly activated pattern of led ON/led OFF (2 s each, 4.5 mW). As a control, an optogenetic single-pulse stimulus was delivered close to the pipette tip, resulting in a fast downward deflection, indicating that ChR2 was effectively transfected and functioning.

#### Pre-processing of imaging data

Images were analyzed with ImageJ and OriginPro (OriginLab, 2017). Frames displaying artifactual excitation of skull autofluorescence were removed (2 out of 3 frames) and interpolated. A daily individual mask was created using the maximum intensity projection of the first imaging session (baseline). Masks were thresholded twice the mean value of the non-transfected hemisphere. For each imaging session, the fluorescence ratio change (ΔF/F_0_) was calculated averaging the first 50 frames before the stimulus onset (baseline fluorescence signal; F_0_).

#### Spatiotemporal propagation analysis

Spatiotemporal propagation analysis was performed with custom-made Python (Python Software Foundation, Beaverton, Oregon, U.S.A.) scripts (Data S1). In a pre-processing step, image sequences were spatially masked and frames containing the laser stimulations were manually eliminated and replaced with their temporal linear interpolation. Then a Gaussian smoothing was performed along the temporal dimension (with a standard deviation for Gaussian kernel equal to one) before computing the ΔF/F_0_ signal. F_0_ was set as the average fluorescence value observed before the first laser stimulus. Pixels were identified as active if the maximum value of the ΔF/F_0_ signal after the first laser stimulus was larger than both the average value and the double of the standard deviation value, computed in both cases before the first stimulus. In active pixels, the time frame corresponding to the first crossing of a pixel-based threshold was used to identify the timing of the response to the laser stimulus. The threshold was set as twice the standard deviation value of the ΔF/F_0_ signal computed before the first laser stimulus. For imaging acquisitions pertaining to CNQX manipulation, the standard deviation values used to identify the active pixels and to define the timing thresholds were computed solely on the data acquired before CNQX administration (vehicle). For the data acquired after CNQX administration, the average of the standard deviations computed before CNQX administration was employed. In all cases, the timing values were then rank-transformed. The rank values of the active pixels related to the same animal and the same condition were averaged and the standard deviation was computed, while the non-active pixel values were discarded. Starting from these averaged results, for data related to CNQX manipulation, rank distributions were computed in a region of interest (ROI) overlapping the LBMMs. The distribution medians before and after CNQX administration were then compared using Wilcoxon signed-rank test. Moreover, to summarize the distribution characteristics, their interquartile ranges were computed alongside the medians. Finally, to trace the propagation direction, for each averaged result, pixels placed along a circumference centered on the laser-stimulated area and with varying radius were selected, discarding non-active or masked pixels. For each circumference radius, the averaged rank distribution was computed and values composing its first quintile were sub-selected. Then the circular mean (Mardia and Jupp, 1999) of the angular position (relative to the circumference center) of these values was computed. Finally, all the computed circular means were used to calculate the final, radius-dependent, circular mean and circular standard deviation (Mardia and Jupp, 1999).

#### Pharmacology

For pharmacological interference experiments, a small craniotomy (∼1/1.5 mm diameter) was performed on the region of interest. For the animals that underwent pharmacological inhibition of both RFA GRASP and TAP, the experiments were performed in a 3-day separate section. Dura was carefully removed to avoid bleeding and the formation of blood clots on the brain surface. The craniotomies were then sealed with Kwik-seal (World Precision Instrument) after the experimental sections. Glutamate receptor antagonist CNQX 1 mM (C127 Sigma-Aldrich) and vehicle (physiological solution containing 0.01% DMSO) were applied to the craniotomy and the solutions were replenished (at the same concentration) every 10′ to compensate for tissue drying. Stimulation sessions were performed every 10’.

#### Immunohistochemistry

Four weeks after injection, mice were perfused with 20–30 mL of 0.1 M PBS (pH 7.6) and 150 mL of 4% paraformaldehyde (PFA). Brain coronal slices (100 μm thick) were cut with a vibrating-blade microtome (Vibratome Series 1500—Tissue Sectioning System). Slices were washed with PBS and incubated in PBS/0.3% Triton X-100 containing 1% BSA for 60 min while shaking at room temperature (RT). Then, slices were washed with PBS/0.1% Triton X-100 (T-PBS) and incubated with the primary antibody NeuN (1:200, Sigma, ABN78) in T-PBS for 1 day at 4°C while shaking. Then, slices were washed with T-PBS and incubated with anti-rabbit fluorescent Alexa 514 antibody (1:250, ThermoFisher, A-31558) in T-PBS for 2 h at RT while shaking. Finally, slices were washed and mounted on a glass slide. Imaging was performed across all cortical layers (II/III and IV) with a confocal laser scanning microscope (CLSM, Nikon Eclipse TE300, with the Nikon C2 scanning head), equipped with a Nikon Plan EPO 60× objective, N.A. 1.4, oil immersion. The setup was equipped with 408 nm, 488 nm and 561 nm lasers to simultaneously excite ChR2, Alexa 514 and jRCaMP1a, respectively. A triple-band dichroic mirror 408/488/543 was used for simultaneous 3-channel fluorescence imaging of both layer II/III and layer V neurons. Emission filters were 472/10 nm, 520/35 nm and 630/69 nm. ChR2 transfection efficiency was not quantified due to the diffuse labeling.

### Quantification and statistical analysis

All statistical analysis was performed in OriginLab (2018) except for the spatiotemporal propagation analysis that was performed with custom-made Python (Python Software Foundation, Beaverton, Oregon, U.S.A.) scripts. Data are shown as mean ± s.e.m. Parametric tests were used only after verifying for normality of the data employing Shapiro-Wilk and Kolmogorov-Smirnov tests. The error bars and shadows in graphs represent the s.e.m. In the box charts, the red line corresponds to the mean, the box shows the standard error range, whiskers lengths are the extreme data points. Student’s t-test was employed for every comparison concerning two samples and its paired version was used for paired data (Figures 7B and 7C; Figures 7B and 7C). For spectral band multiple comparisons in Figure S1B, two-way (variables: illumination status and bands) ANOVA was used. For multiple comparisons in Figures 6B, 6E, and 6F (for onset, distance and elevation values, respectively) and in Figure S5A (for average calcium transients), one-way ANOVA was used and Bonferroni correction was applied for post-hoc t-tests. The level of significance was set at ^∗^p < 0.05, ^∗∗^p < 0.01, and ^∗∗∗^p < 0.001.

## Data Availability

Data reported in this paper will be shared by the lead contact upon request. All original code is available in this paper’s supplemental information, named: Data S1 propagation analysis script. Related to Figure 5. Any additional information required to reanalyze the data reported in this paper is available from the lead contact upon request.

## Supplemental information

Document S1. Figures S1–S5

Data S1. Propagation analysis script, related to Figure 5

Document S2. Article plus supplemental information
