# Supplementary material for: Large-scale all-optical dissection of motor cortex connectivity shows a segregated organization of mouse forelimb representations
Source: Cell Rep. 2022 Nov 8;41(6):111627. doi: 10.1016/j.celrep.2022.111627 (PMC10073205; doi:10.1016/j.celrep.2022.111627)
Supplement: Document S1. Figures S1–S5 [file mmc1.pdf]

Cell Reports, Volume *41*

Supplemental information

Large-scale all-optical dissection of motor cortex connectivity shows a segregated organization of mouse forelimb representations

Francesco Resta, Elena Montagni, Giuseppe de Vito, Alessandro Scaglione, Anna Letizia Allegra Mascaro, and Francesco Saverio Pavone

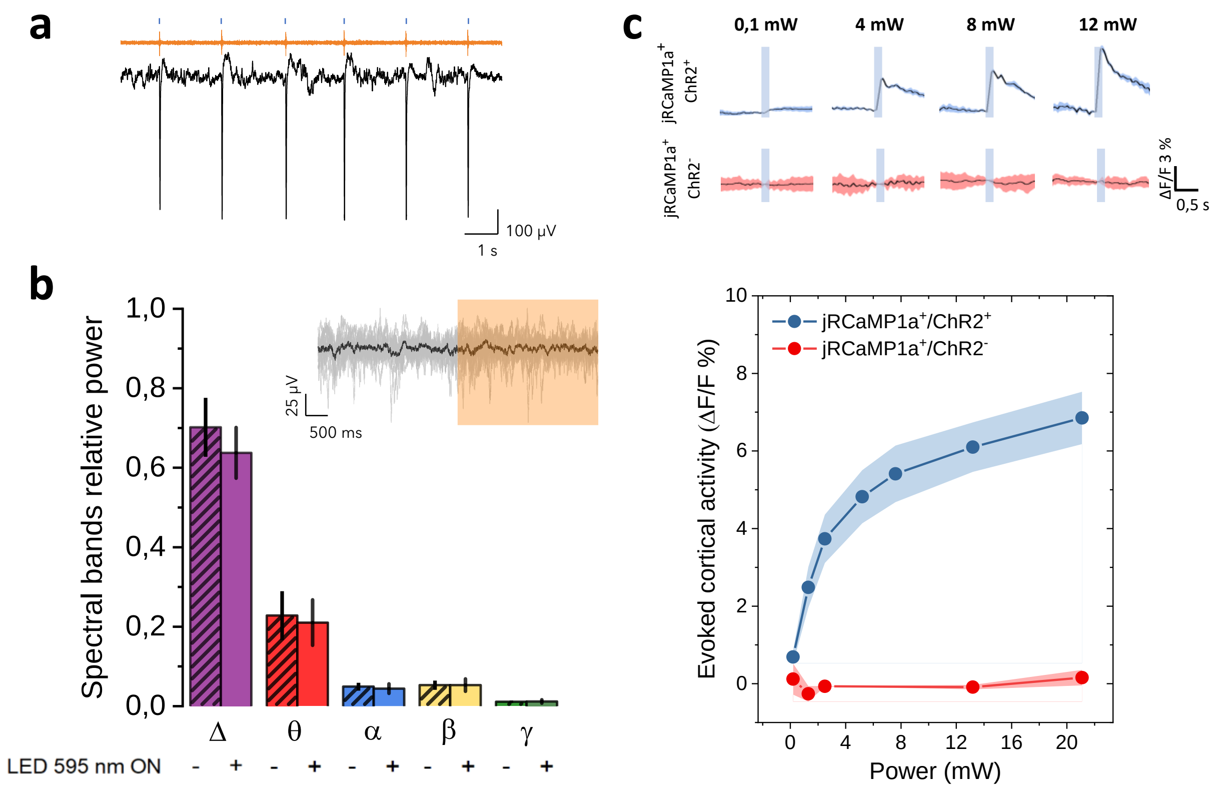

Supplementary fig.1 Wide-field imaging of jRCaMP1a does not induce ChR2 cross-activation. Related to Figure 1.

(a) Representative trace of the LFP response to optogenetic stimulation. Blue tags represent laser pulses (10 ms), the orange trace represents the high-pass filtered LFP signal (200-1000 Hz) and the black line represents the LFP signal (0.5-100 Hz). (b) Quantification of the spectral band relative power during 6 s of peri-stimulus period (n = 4 mice, 10 stimuli per mice). Columns represent the averaged relative power for LFP frequency bands (Δ; θ; α; β; γ) during the dark period (-3 - 0 s; gray) and the LED illumination period (0 - +3 s, orange; nmice = 4, 10 per mouse; two-way ANOVA with post-hoc Bonferroni test; Data are presented as mean ± SEM). Inset: representative traces of the LFP signal (gray lines; n = 10; black line = average; LED on period in orange). (c) Top panel. Representative traces showing the average calcium response (black) and the SEM (shadows) at increasing laser power (0,1 – 10 mW; 20 ms laser illumination) in mice expressing jRCaMP1a + ChR2 (blue) and only jRCaMP1a (red). Bottom panel. Correlation between evoked calcium activity and single pulse laser power in mice expressing jRCaMP1a^{+}/ChR2^{+} (blue; n = 8) and jRCaMP1a^{+}/ChR2^{-} (red; n= 2). Shadows represents SEM.

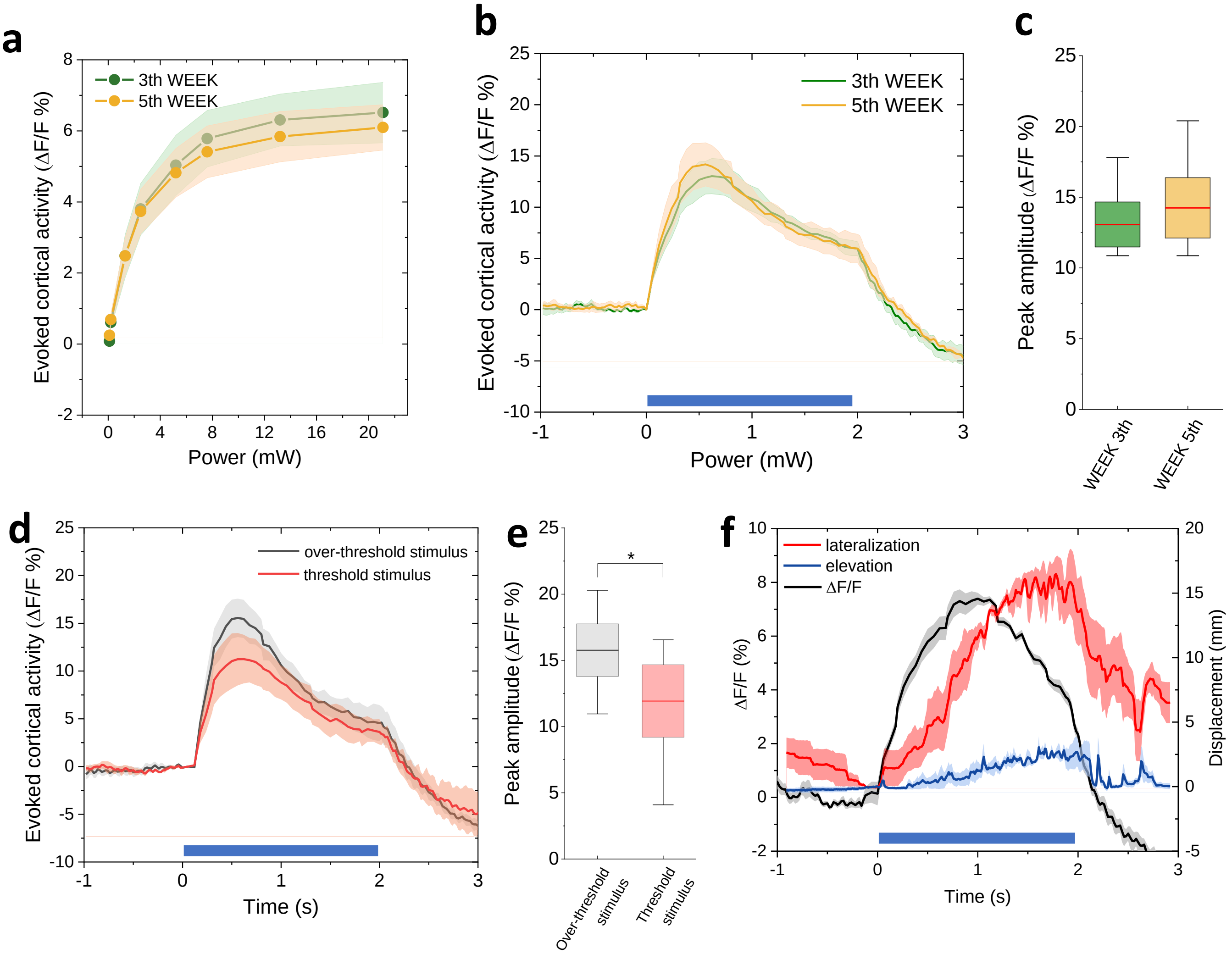

Supplementary fig.2 Optogenetically-evoked calcium transients show long-term stability and no ceiling effects ad the minimum laser power requires to evoke movements. Related to Figure 2.

(a) Correlation between single laser pulse (10 ms) intensities and the calcium transient responses 3 and 5 weeks post-infection. (b) Calcium transients evoked by optogenetic stimulus trains (20 ms, 16 Hz; 2s) showing long-term profile stability. Bottom blue line represents the stimulation period. (c) Quantification of the stimulus train-evoked calcium transient amplitudes (d) Calcium transients evoked by optogenetic stimulus at the threshold laser power required to evoke movement (red) and using an over-threshold stimulus (black). Bottom blue line represents the stimulation period. (e) Quantification of the calcium transient amplitude evoked by over-threshold (black) or threshold stimulus (red) (n=6). (f) Image showing the relationship between the forelimb position in terms of elevation (blue) and lateral displacement (red) with the calcium transient signal recorded in the site of stimulus (black) during an optogenetically-evoked GRASP movement (n animal = 1; n train = 5). Dark traces represent the average and shadows represent SEM. Bottom blue line represents the stimulation period.

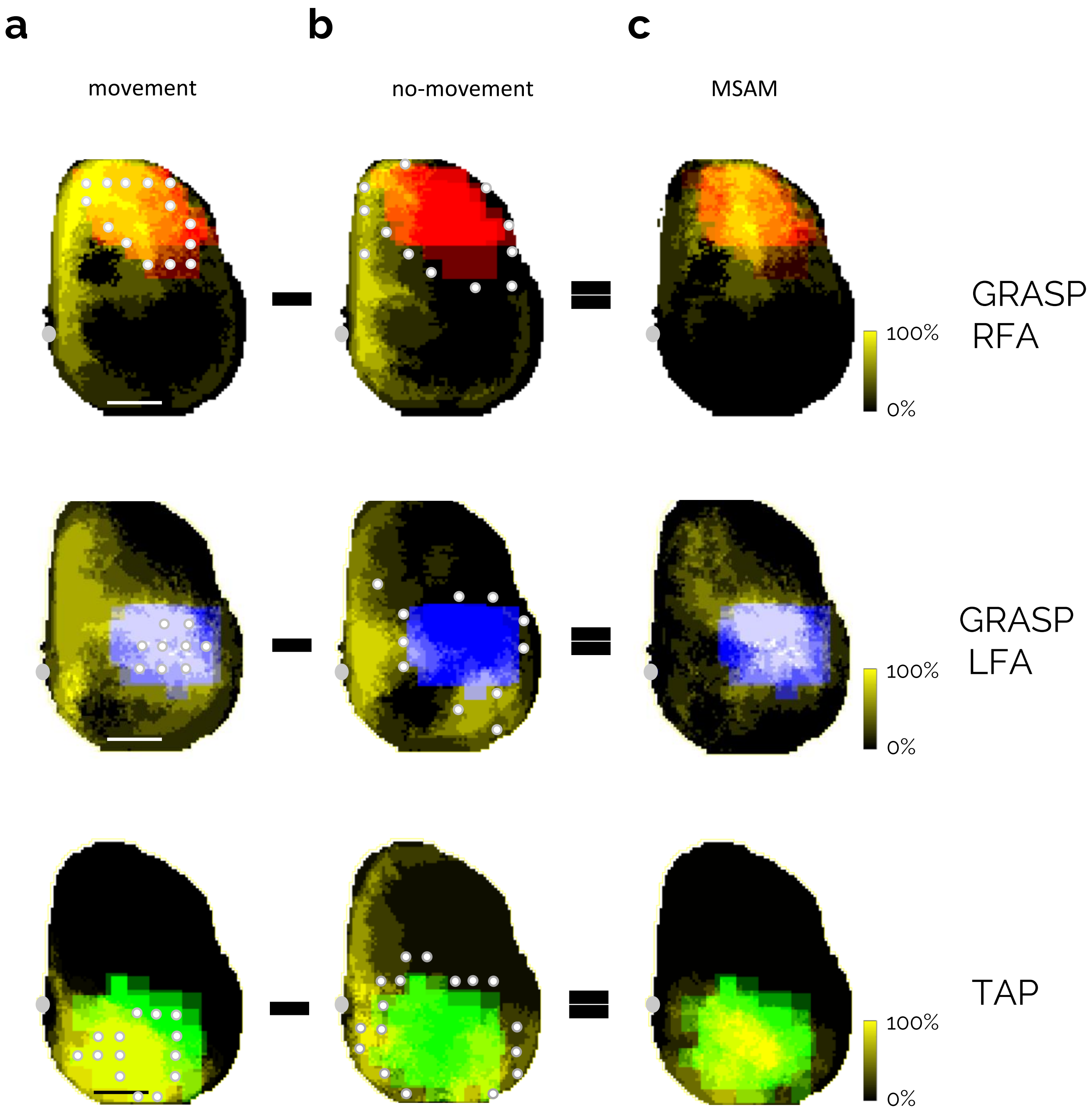

Supplementary fig.3 Movement-specific activation maps (MSAMs) processing. Related to Figure 3-4.

Average activation maps (yellow) obtained by selectively stimulating single points (white dots) within the LBMM (colored areas) (a) and outside the LBMM (b). (c) Representative movement-specific activation maps (MSAMs) obtained by subtracting maps obtained in (b) to those obtained in (a).

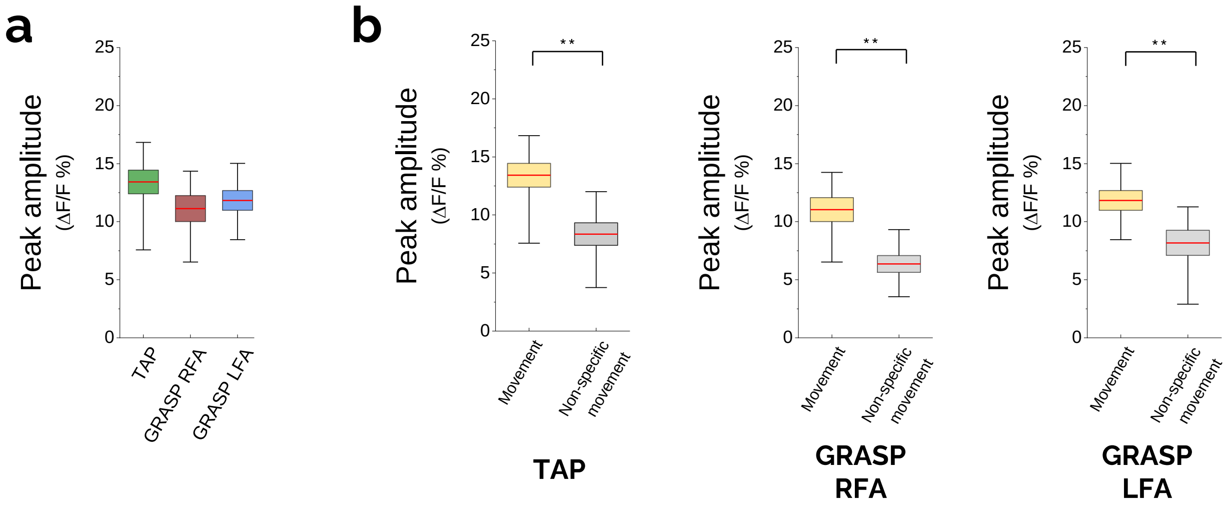

Supplementary fig.4 Comparison of average calcium transient properties between movement categories. Related to Figures 3 and 4.

(a) Comparison of evoked calcium transient amplitudes between movement categories extracted from a ROI placed over the site of stimulation (TAP 13.4 ± 1.0; GRASP RFA 11.1 ± 1.1 ΔF/F; GRASP LFA 11.8 ± 0.8 ΔF/F; n = 7, one-way ANOVA with post hoc Bonferroni test) (b) Comparison of the ROI calcium transient amplitudes obtained stimulating within the LBMM (Movement) and outside the LBMM (non-specific movement) per movement classes (TAP: Movement = 13.4 ± 1.0 ΔF/F vs Non-specific movement 8.3 ± 0.9 ΔF/F; GRASP RFA: Movement = 11.1 ± 1.0 ΔF/F vs Non-specific movement 6.3 ± 0.7 ΔF/F; GRASP LFA: Movement = 11.8 ± 0.8 ΔF/F vs Non-specific movement 8.1 ± 1.0 ΔF/F; n = 7, ** p<0,01 two sample t-test).

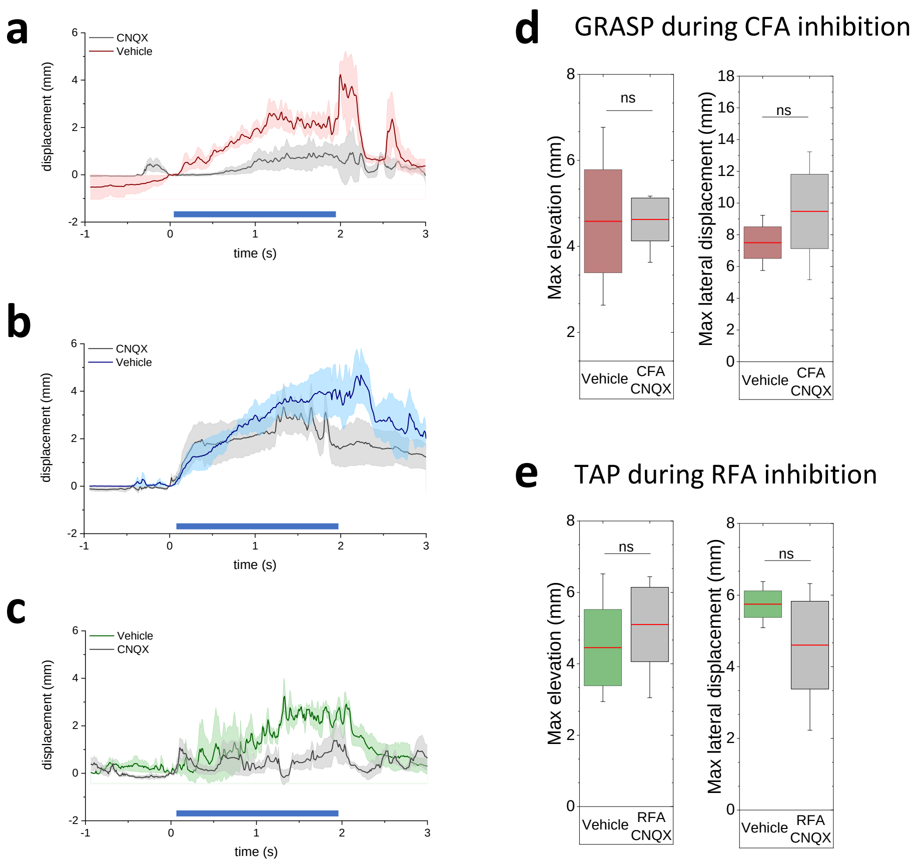

Supplementary fig.5 Excitatory synaptic transmission block disrupts only the related complex forelimb movement kinematics. Related to Figures 7 and 8.

(a) Forelimb elevation displacement during GRASP RFA before and after CNQX topical application (n = 3). Blue line indicates stimulation period. Dark traces represent the average and shadows represent SEM. (b) and (c) show the same analysis for GRASP LFA and TAP respectively. (d) Effect of CFA CNQX topical application on GRASP kinematics. Comparison of the absolute left forelimb maximum elevation (left) and maximum lateral displacement (right) following RFA stimulation in vehicle and after CFA CNQX topical application (n = 3, paired sample t-test). Red lines indicate means, boxes show the standard error range, whiskers length represents the extreme data points. (e) Effect of RFA CNQX topical application on TAP kinematics. Comparison of the absolute left forelimb maximum elevation (left) and maximum lateral displacement (right) following CFA stimulation in vehicle and after RFA CNQX topical application (n = 3, paired sample t-test). Red lines indicate means, boxes show the standard error range, whiskers length represents the extreme data points.
